# Pre-selection of banana somaclones resistant to *Fusarium oxysporum* f. sp. *cubense*, subtropical race 4

**DOI:** 10.1016/j.cropro.2021.105692

**Published:** 2021-09

**Authors:** Tamyres Amorim Rebouças, Anelita de Jesus Rocha, Tamires Sousa Cerqueira, Poliana Ramalho Adorno, Rafael Queiroz Barreto, Mileide dos Santos Ferreira, Lucymeire Souza Morais Lino, Vanusia Batista de Oliveira Amorim, Janay Almeida dos Santos-Serejo, Fernando Haddad, Claudia Fortes Ferreira, Edson Perito Amorim

**Affiliations:** a Universidade Estadual de Feira de Santana, Feira de Santana, Bahia, Brazil; b Universidade Federal do Recôncavo da Bahia, Cruz das Almas, Bahia, Brazil; c Embrapa Mandioca e Fruticultura, Rua Embrapa, s/n CP 007, Chapadinha, Cruz das Almas, Bahia, Brazil

**Keywords:** *Musa* species, Genetic improvement, Somaclonal variation, Plant health

## Abstract

– Fusarium wilt, caused by *Fusarium oxysporum* f. sp. *cubense* (Foc), is one of the most destructive diseases affecting banana crops worldwide. Therefore, the development of resistant cultivars is a promising alternative to mitigate the effects of the disease on banana plantations. The objectives of this study were to induce somaclonal variation in banana cultivars of the Silk and Cavendish types and to select somaclones resistant to subtropical race 4, thereby enabling the production of fruit in areas where this race is present. Shoot clump apexes of the Grand Naine and Maçã (Silk) cultivars were grown in MS medium. The cultures were subcultured four times. They were then challenged with fusaric acid (FA) in an experiment consisting of four treatments with different concentrations (0.1, 0.2, 0.3, and 0.4 mM) and five repetitions, each consisting of a Petri dish containing seven multiple shoot clumps in MS culture medium supplemented with 2.5 mg/L benzylamine purine. Multiple shoot clumps without the addition of FA were also used in the experiment, and were subcultured three times and maintained in a dark room. The multiple shoot clumps that survived the treatment with FA were transferred to MS medium and maintained in the growth chamber in the presence of light. The regenerated plants were later planted in tanks containing soil infested with an isolate classified as Foc subtropical race 4 (Foc STR4), and were evaluated for resistance to the pathogen at 90 days after inoculation (d.a.i.). Pathogen structures were confirmed by root clarification and root staining technique. All somaclones of the Maçã (Silk) cultivar were susceptible to Fusarium wilt and two somaclones of the Grand Naine cultivar were selected as resistant. The addition of FA as a selective agent was effective in the selection of somaclones among plants of the Grand Naine cultivar, as shown by the selection of two somaclones resistant to Foc STR4. The next step will consist of the agronomic and market potential validation of the selected somaclones, aiming to confirm their potential use by producers.

## Highlights

• Fusarium wilt most destructive disease affecting banana worldwide.
• Resistant cultivars is a promising alternative.
• Somaclonal variation is an interesting tool for breeding.
• Two somaclones of the Grand Naine were selected as resistant.
• Next step will consist of the agronomic and market potential validation of the selected somaclones.

## 1 Introduction

Bananas are one of the most consumed fruits and is a staple for millions of people around the world (Maymon et al., 2020). India is the world's largest producer, with 30.8 million tons produced in 2019 followed by China and Indonesia with 11.5 and 7.2 million tons, respectively. Brazil ranks fourth, with production of approximately 7.0 million tons (FAOSTAT, 2021). As other commercial crops, banana production is affected by diseases, especially those caused by fungi that lead to great losses in fruit quality and production. These include yellow Sigatoka (*Pseudocercospora musicola*, Leach), black Sigatoka (*Pseudocercospora fijiensis*, Morelet), and Fusarium wilt (*Fusarium oxysporum* f. sp. *cubense*) (Ploetz, 2015; Alakonya et al., 2018; Santos et al., 2019).

Fusarium wilt, caused by *Fusarium oxysporum* f. sp. *cubense* (Foc), is considered one of the most destructive diseases affecting banana crops and caused one of the worst plant disease epidemics. The pathogen is present in practically all banana producing areas in the world. It is a soil-borne fungus that produces resistance structures called chlamydospores, which allow its survival in the soil for decades (Ploetz, 2015). The process of infection begins when the pathogen penetrates the banana roots and colonizes the vascular system of the plant, thereby hindering the transport of water and nutrients to the aerial parts. The external symptoms begin with the yellowing and wilting of older leaves, which then progresses to the younger leaves. Internal symptoms include the discoloration, browning and necrosis (Dita et al., 2018).

Fusarium wilt first caused significant damage to Gros Michel bananas in Central America in the 1900s, responsible at that time for the global banana export trade worldwide (Stover, 1962; Buddenhagen et al., 2009). As a result, Gros Michel was replaced by the Cavendish cultivars, for being resistant to Foc race 1. However, in 1980, a new race of pathogenic Foc - to the Cavendish subgroup cultivars spread throughout Asia, Africa, Indonesia, and South America, and was named tropical race 4 (TR4) (Stover, 1962; Buddenhagen et al., 2009; Dita et al., 2018; Garcia-Bastidas et al., 2019). Due to its interaction with Cavendish bananas under different environmental conditions, Foc race 4 was subdivided into “tropical” (TR4) and “subtropical” (STR4) (Ploetz, 2006a). The subgroup TR4 is highly pathogenic and infects bananas regardless of environmental conditions (Buddenhagen, 2009). According to Ploetz (2006b), stress, such as low temperatures, may lead to STR4 symptoms in banana plants.

Thus, Foc has been classified into three races, based on the range of susceptible hosts, and also into vegetative compatibility groups (VCGs) (with a total of 24 groups identified) (Mostert et al., 2017). The three main races that affect dessert bananas are as follows: race 1, which affects the Gros Michel and Lady Finger cultivars; race 2, which affects the same cultivars as race 1 and also the Bluggoe cultivar; and race 4, which causes disease in most cultivars, including the Cavendish cultivar (Ploetz, 2015).

Control measures, including crop management practices, such as keeping soil pH over 6.0 and biological control, have been used to minimize the impacts of the disease; however, they are not effective in controlling the disease, especially in areas with high inoculation pressure (Haddad et al., 2018). In this context, the use of resistant cultivars is the most effective option to mitigate the negative impacts of the pathogen in banana production fields (Dita et al., 2018; Haddad et al., 2018; Rebouças et al., 2018).

Obtaining resistant cultivars through genetic breeding has been successful., Hybrids resistant to race 1 have been developed at the breeding programs of the Honduras Foundation for Agricultural Research (FHIA) and the Brazilian Agricultural Research Corporation (EMBRAPA), among other breeding programs ((Amorim et al., 2013; Khayat and Ortiz, 2011; Rocha et al., 2021). The main obstacle in the process of conventional banana breeding is the sterility of some cultivars, especially triploid species, which leads to low seed production in crosses, making it difficult to transfer characteristics of interest from improved diploid species to commercial cultivars (Silva et al., 2013). It is also noteworthy that the taste of banana hybrids developed by conventional breeding is not always acceptable nor palatable to consumers.

The use of biotechnology tools has allowed advances in banana genetic studies, especially through techniques such as genetic engineering, induction of mutations, somaclonal variation, somatic hybridization, and polyploidy, which can be used to overcome the barriers of natural sterility found in some cultivars (Pestana et al., 2011; Amaral et al., 2015; Borges et al., 2016; Rocha et al., 2021). Among the mentioned tools the induction of somaclonal variations has been recognized as important for generating genetic variability as an alternative for banana genetic improvement, which allows the selection of somaclones with the desired characteristics (Ghag et al., 2014). The selection of somaclones in species with vegetative propagation such as bananas allows large scale multiplication of superior genotypes maintaining their genetic identity and with quick access by producers (Amorim et al., 2021).

Somaclonal variation is caused by subjecting the plant to stress under *in vitro* growing conditions, which can generate disturbances during cell division and thus lead to genetic or epigenetic variations (Nwauzoma; Jaja, 2013). Number of subcultivation cycles or the effects of hormones or plant growth regulators contribute to natural somaclone variant formation *in vitro* cultivation. Furthermore, the induction of somaclonal variation can also be associated with the use of mutagenic agents in genetic breeding (Deepthi e Phani, 2018; Jain, 2010). Changes of genetic origin are inherited by subsequent generations and epigenetic changes correspond to transient variations due to the physiological stress suffered *in vitro* (Penna et al., 2019; Anil et al., 2018).

Somaclonal variation induction in bananas were successful in generating Fusarium resistant somaclones. The Taiwan Banana Research Institute (TBRI) has induced somaclonal variation in banana of the Cavendish subgroup and identified plants resistant to tropical race 4 (TR4), such as the GCTCV-218 somaclone, registered for commercial cultivation under the name Formosona (Hwang and Ko, 2004; Molina, 2016). Another study was conducted at the Bhabha Atomic Research Center (India), in which the authors subcultivated somatic banana embryos for a period of 14 years and obtained 26 somaclones. These were tested with Foc race 1 inoculum and four somaclones of the Rasthali cultivar that were resistant to the pathogen, were selected (Ghag et al., 2014).

Selective agents are frequently used in culture media to offer selection pressure *in vitro* in somaclonal plants in order to hasten the sorting out of plants that were submitted to mutation for desired traits (Jain, 2010; Flores e Bruckner, 2014). An example of a chemical agent used in tests, which confer resistance to diseases, is fusaric acid, an unspecific toxin produced by many species of fungi from the genus *Fusarium*. Efficient use of this toxin for *in vitro* selection of banana plants resistant to Foc race 1 was used in the Silk (Maçã) cultivar (Matsumoto et al., 1995) e cv. Rasthali (Saraswathi et al., 2016).

Therefore, the aims of this study were to induce somaclonal variation in banana cultivars of the Silk (Maçã) and Cavendish types through successive cultivations and supplementation of the culture medium with plant regulators and to select potential somaclonal variants by means of *in vitro* selection with fusaric acid for the production of new banana genotypes resistant to Fusarium wilt.

## 2 Materials and methods

The experiments were carried out at the Plant Tissue Culture Laboratory and in a greenhouse at Embrapa, located in Cruz das Almas (12°40′19″S, 39°06′22″W', altitude 220 m above sea level), Bahia, Brazil. The climate is classified as hot humid tropical, Aw to Am, according to the Köppen classification, with a mean annual temperature of 24.5 °C, relative humidity of 80%, and mean annual rainfall of 1250 mm (AGRITEMPO, 2018).

### 2.1 *In vitro* production of multiple clump shoot from shoot apexes

Multiple shoot clump induction was performed using shoot clump apexes of the cultivars Grand Naine (AAA, subgroup Cavendish) and Silk (Maçã) (AAB, subgroup Silk) cultivated *in vitro* in MS medium (Murashige and Skoog, 1962). The shoot clump apexes were grown in an MS medium consisting of salts and vitamins, supplemented with 1 mg/L paclobutrazol® (PBZ), 1 mg/L thidiazuron (TDZ), 1.6 mg/L indole-3-acetic acid, 80 mg/L adenine hemisulfate, and 30 g/L sucrose, with the pH adjusted to 5.8 and solidification with 2.4 g/L phytagel®. The explants were cleaned by removing the oxidized parts and layers of the leaf sheaths to expose the shoots, which were then cut longitudinally at the meristematic end to break the apical dominance and stimulate shoot multiplication.

### 2.2 *In vitro* pre-selection with fusaric acid

**Fig. 1:**
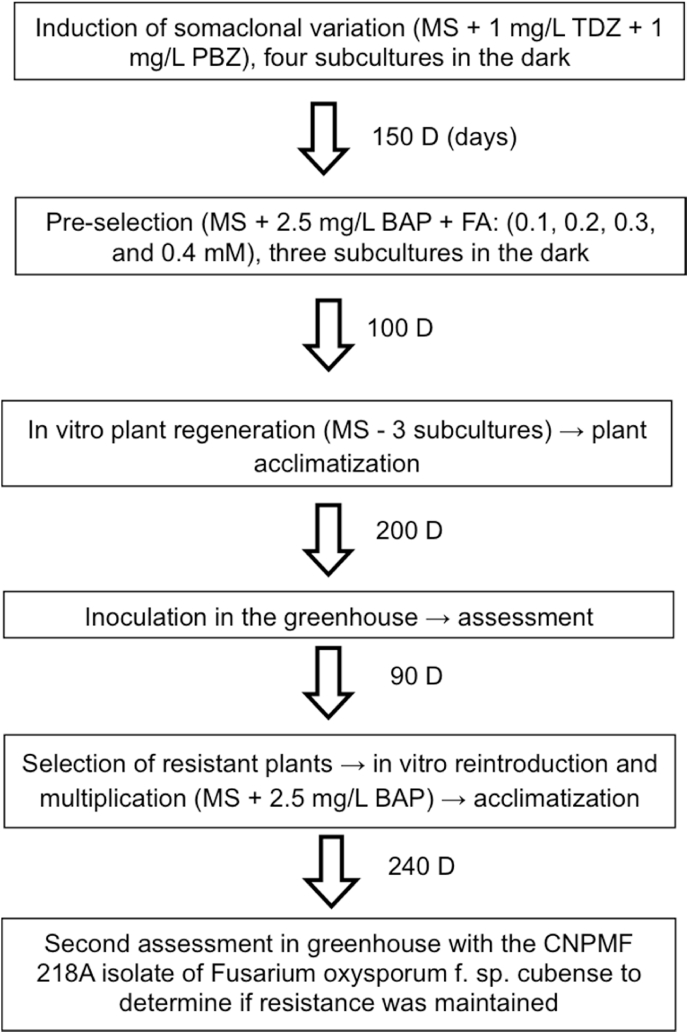
Flowchart of the selection of Grand Naine and Maçã (Silk) cultivar plants resistant to Fusarium wilt. TDZ: thidiazuron; PBZ: paclobutrazol®; BAP: benzylaminepurine; FA: fusaric acid. Embrapa, Brazil, 2021.

**Fig. 2:**
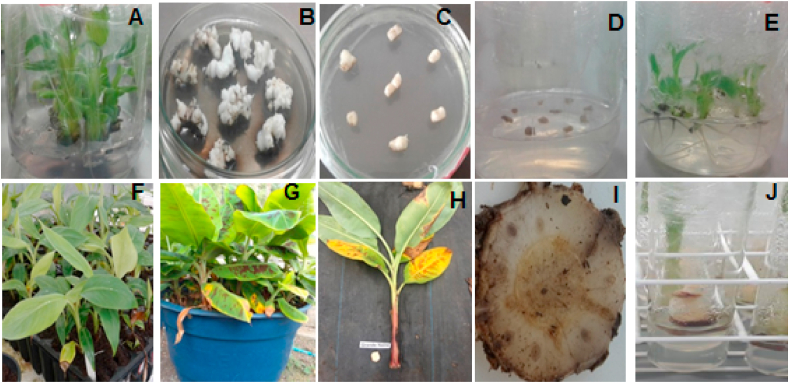
Stages of the *in vitro* selection of somaclones. *In vitro* micropropagated plants (A), induction of multiple bud clumps (B), multiple shoot clumps cultured in fusaric acid-containing medium (C), survival and regeneration of plants from multiple shoot clumps after fusaric acid treatment (D and E), acclimatization (F), planting in soil infested with the CNPMF 218A isolate representative of subtropical race 4 (G), cutting of plants for analysis of external (H) and internal (I) symptoms, and reintroduction of resistant plants to tissue culture (J). Embrapa, Brazil, 2021.

The shoot clumps from the Grande Naine and Silk (Maçã) were submitted to the selected agent fusaric acid (FA) after four subcutivations in culture medium in order to induce somaclonal variation. Therefore, the experiment consisted of four treatments with different concentrations of FA (Sigma-Aldrich, EUA) (0.1; 0.2; 0.3; and 0.4 mM), with five replicates, each replicate represented by a plate containing seven multiple shoot clumps in the MS culture medium supplemented with 2.5 mg/L benzylaminepurine (BAP). A completely randomized design was used. Multiple shoot clumps without the addition of FA were also used in the experiment. Multiple shoot clumps were subcultured three times at intervals of 30–40 days and kept in a dark room. At the end of the third subculture, the multiple shoot clumps that survived the treatment with FA were transferred to MS medium and kept in the growing room in the presence of light (light intensity: 36 μmol m^−2^ s^−1^, photoperiod: 16 h light) and at a temperature of 25 ± 2 °C for plant regeneration. The regenerated plants were transplanted into tubes containing a commercial substrate of Plantmax coconut fiber (PlantMax, Brazil) and acclimatized for 60 days in the greenhouse (Fig. 1, Fig. 2).

### 2.3 Foc isolate and cultivation conditions

For the resistance tests, isolate CNPMF 218A, a Foc STR4 isolate, was used. The culture used belongs to the biological collection of the phytopathology Laboratory at Embrapa Mandioca e Fruticultura. This isolate was collected in the state of São Paulo where it affects the Nanica (Cavendish) cultivar. Studies performed with this isolate showed that it was virulent and aggressive in causing symptoms of Fusarium wilt in Grand Naine; a cultivar belonging to the Cavendish subgroup and resistant to Foc race 1 after periods of stress dueto harsh winters and/or water deficit (Haddad et al., 2011; Costa et al., 2015). This CNPMF 218A isolate was characterized as subtropical race 4 (STR4), belonging to the vegetative compatibility group (VCG) 0120 based on tests of VCGs (Rocha et al., 2020). Twenty-four Foc VCGs are known, of which VCG 0120, 01201, 01202, 01209, 01210, 01211, 01215, and 0120/15 are associated with Foc STR4, and only two VCG, VCG 01213/16 and 0121, was reported for Foc TR4 (Aguayo et al., 2017; Buddenhagen, 2009; Dita et al., 2018). The Maçã (Silk) and Grand Naine cultivars were selected for testing because of their susceptibility levels to this race of Foc.

The inoculum was produced from cuttings from isolate CNPMF 218A in plates containing potato dextrose agar (PDA) incubated in BODs with 25 °C temperature and 12 h photoperiod. After colony growth a conidia suspension from the isolate was prepared. Twenty microliters (20 mL) of this suspension was sown in 1 kg of properly sterilized rice. The culture medium was incubated in the BOD at 25 °C temperature and 12-h photoperiod.

After 20 days a serial dilution of the infested rice was prepared and the colony formation unit (CFU) counted in order to adjust the concentration and viability of spores. The CFUs were counted using the Neubauer chamber and concentration adjusted to a 10^6^ CFU/g of substrate for inoculations (Madigan et al., 2016).

### 2.4 Preliminary assessment of somaclones in the greenhouse

The “Silk” (Maçã) and “Grand Naine” plants recuperated in the pre-selection with FA were massively multiplied in MS and acclimatized in the greenhouse for 45–60 days and at 15 cm transferred into polyethylene boxes containing soil infested with the isolate CNPMF 218A (10^6^ CFU/g of solo).

The internal symptoms of rhizome discoloration were evaluated after 90 days or after the death of the plant, according to the scale proposed by Dita et al. (2014), with scores ranging from 1 to 5, where: (1) no symptoms; (2) initial rhizome discoloration; (3) slight rhizome discoloration; (4) rhizome with most internal tissues showing necrosis; (5) totally necrotic rhizome. Based on the scores of the internal symptoms of the evaluated plants, the frequency of each score was calculated and converted to a percentage.

### 2.5 Confirmation of resistance of somaclones in the greenhouse

The resistant somaclones that did not present symptoms of Fusarium wilt in the first test were reintroduced *in vitro* through cuttings of the explants removing part of the rhizome and pseudostem until reaching pieces of 5 cm (height) x 1,5 cm (diameter) obtaining a cylinder formed by the stem apex. These were then disinfested and established *in vitro* in MS medium culture with salts and vitamins (Murashige e Skoog, 1962) and cultivated for 15 days under dark conditions and 15 days in the growth chamber under light with a 16 h photoperiod and photon flux density of 30 μEm^−2^s^−1^, a 27 ± 2 °C. Afterwards, the explants were transferred and multiplied in MS + 2.5 mg/L of BAP to obtain a higher number of clones, allowing a second assessment of the resistance to Foc STR4 using the same method as stated in the first stage, whereas only ten replicates per somaclone (*in vitro* cloned plants), were used.

Ninety days after planting, the internal symptom scores were translated into the disease intensity index (DI) according to the formula described by McKinney (1923). ID data were transformed into log (x+1) in order to be in compliance with the analysis of variance and afterwards the averages were grouped by the Scott-Knott test with 5% probability using the Exp.Des.pt package in the R software (R Core Development Team, 2017).

### 2.6 Clearing and staining of banana roots

Clearing and staining of the roots was performed according to the method proposed by Phillips and Hayman (1970) to visualize the fungal structures in the plant samples, using 1–2 cm long root fragments collected from the somaclones that were identified as resistant to STR4 Foc.

Clearing was performed by immersing the cuttings of the root system in a 10% KOH solution placed in a water bath at 90 °C for 1 h. Then, the KOH was discarded, and the samples were washed with water to completely remove the solution and transferred to a 1% HCl solution for 5 min. Subsequently, they were stained with a 0.05% Trypan blue solution heated in a microwave for 40 s, and the dye was then discarded and the roots were immersed in a lactoglycerol solution (2:1:1 of lactic acid, glycerol, water). The root fragments captured via microphotographs taken under a light microscope.

## 3 Results and discussion

### 3.1 *In vitro* pre-selection with fusaric acid

**Fig. 3:**
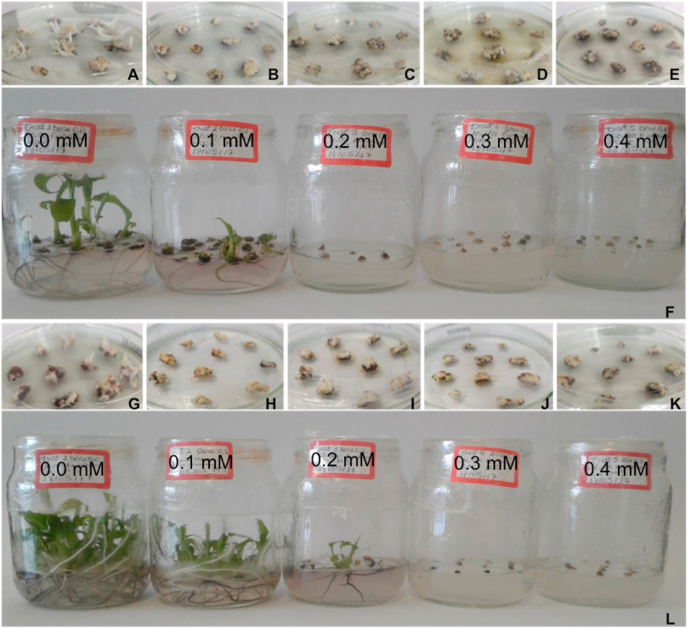
Effect of fusaric acid (FA) doses on plant survival and regeneration using multiple bud clumps of Maçã (A–F) and Grand Naine (G–L) cultivars grown in a medium containing different concentrations of FA. Control treatment (0.0 mM); Treatment 1 (0.1 mM FA); Treatment 2 (0.2 mM); Treatment 3 (0.3 mM); Treatment 4 (0.4 mM). Embrapa, Brazil, 2021.

For the possible somaclones generated in this work survival of the shoot clumps decreased with the increase of fusaric acid (FA) concentration for the cultivars Grand Naine and Silk (Maçã) during *in vitro* pre-selection containing FA (Fig. 3). . Therefore, the generation of the shoot clumps after three subcultivations with FA was obtained only in the control treatments (0.0 mM) and 0.1 mM of FA for the Silk (Maçã) cultivar (Fig. 3F) and control treatment 0.1 mM, and 0.2 mM of FA for of the Grande Naine cultivar (Fig. 3L).

According to Singh et al. (2017), moderate doses of FA (50–100 μM) induce apoptosis (programmed cell death), while high doses of FA (>200 μM) promote necrosis. Thus, when FA is used at low concentrations it can trigger defense reactions in the plant, as it induces the production of reactive oxygen species (ROS), which activates proteins that regulate programmed cell death and other hypersensitivity response elements in plants (Samadi and Behboodi, 2006).

The number of multiple shoot clumps regenerated for the Silk (Maçã) cultivar was nine for the control and six for the 0.1 mM FA treatment. For the 0.2 mM, 0.3 mM and 0.4 mM FA treatments, all multiple shoot clumps showed symptoms of necrosis and no regeneration of plants (Fig. 3F). A higher number of multiple shoot clumps was seen for “Grand Naine” with 56 for the control, 38 for the 0.1 mM and six for the 0.2 mM treatment. Likewise for Silk cultivar, there was no regeneration for the 0.3 mM and 0.4 mM FA treatments for “Grand Naine”. In both cases, the multiple shoot clumps in the control treatment were also selected because they were treated with cultivation medium containing PBZ and TDZ, which indicate they are also potential somaclones.

Differences regarding survival of multiple shoot clumps between the Silk and Grand Naine cultivars can be due to the success in the obtainment of somaclonal variation which confers resistance to Fusarium wilt and further more, studies show that the lethal doses of the toxin seem to vary according to the agent, genotype and race of the pathogen (Saraswathi et al., 2016).

In the study by Matsumoto et al. (1995), the banana cultivars Silk (Maçã) and Nanicão (Cavendish) were tested for tolerance to race 1 and the growth of multiple shoot clumps of both cultivars was completely inhibited in the medium supplemented with 0.1 mM FA. . There was no significant difference between the susceptible Silk (Maçã) and tolerant (Nanicão) cultivars at the highest concentrations of FA; this fact is in agreement with the present results. Moreover, according to these authors, the tolerance of Nanicão to race 1 may not be related to tolerance to FA.

The high phytotoxicity of FA at high concentrations is mainly related to its cell death inducing effect, which directly interferes with the photosynthetic rate of plants. In tomatoes, the application of a *Fusarium oxysporum* f. sp. *lycopersici* culture filtrates directly on the leaves showed that FA contributes to the development of wilt symptoms and has the potential to induce cell death in tissues due to the oxidative explosion caused by the rapid production of ROS (Singh et al., 2017).

In the study by Liu et al. (2020), a FUB gene deletion mutant, related to fusaric acid, was inoculated in the Brazilian type Cavendish (AAA) banana, resulting in the reduction of Fusarium wilt symptoms, which confirmed that FA is associated with the level of virulence of Foc TR4. These authors also reported that leaves treated with high concentrations of FA exhibited necrotic lesions on the surface caused by high ROS production.

### 3.2 Preliminary screening of somaclones resistant to Fusarium wilt under greenhouse conditions

**Fig. 4:**
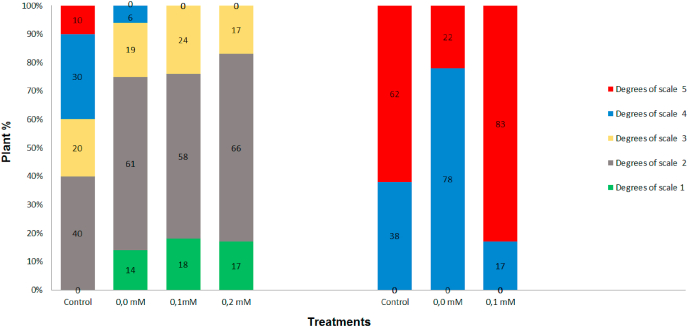
Percentage of somaclones originated from fusaric acid selection of the cultivars Grand Naine (A) and Maçã (B), control (cultivars without induction somaclonal variation) and Concentrations of FA (0.0 mM); (0.1 mM FA); (0.2 mM); (0.3 mM); (0.4 mM). Evaluated 90 days after inoculation with the CNPMF 218A isolate of *Fusarium oxysporum* f. sp. *cubense* according to the scale proposed by Dita et al. (2014): no symptoms (score 1) initial rhizome discoloration (score 2); slight rhizome discoloration (score 3); rhizome with most internal tissues showing necrosis (score 4); totally necrotic rhizome (score 5). Embrapa, Brazil, 2021.

There was no genetic variability as to the response to Fusarium wilt for the somaclones from the Silk cultivar under greenhouse conditions since disease symptoms with high scores (4 and 5) were noted for all plants, including those submitted to the 0.1 mM of FA (Fig. 4B). For the control and the somaclones from the 0.1 mM FA treatment, most internal tissues of the rhizome had some necrosis (score 4) with percentages of 78% and 17%, respectively and the percentage of somaclones with total necrotic rhizomes (score 5) was 22% and 83%, respectively (Fig. 4B).

Although it was possible to select ‘Silk' somaclones at 0.1 mM FA under controlled conditions, this is probably the not best pre-selection option *in vitro* due to its high susceptibility to many Foc races and there are changes related to the FA LD50 according to the pathogenic variability amongst the genus *Fusarium* since this is a virulence strategy adopted by the pathogen (Kuanar et al., 2014; Ramírez-Mosqueda et al., 2015). This justifies the absence of the expected resistance reaction of the somaclones of this cultivar under greenhouse conditions. This difference can also be proven by reports from Matsumoto et al. (1995), which selected ‘Silk' plants tolerant to Fusarium using 0.1 mM of FA after chemical treatment with mutagenic agents whereas the authors used a race 1 isolate with inferior pathogenicity compared to STR4.

In this case, a feasible strategy for using FA as a pre-selection agent is to add a filtrate of pure Foc culture to the plant growth medium instead of the synthetic toxin of the pathogen. This strategy was efficient for selecting somaclones resistant to *F. oxysporum* f. sp. *zingiberaceae,* which showed that continuous *in vitro* selection by the addition of toxins, pure Foc culture filtrate or pathogens to the growth medium increases the frequency of resistant plants (Kuanar et al., 2014).

For the Grand Naine cultivar the somaclones from the control, 0.1 mM and 0.2 mM of FA, presented 14%, 18%, and 17% of plants without symptoms of the disease (score 1); 61%, 58%, and 66% with initial discoloration of the rhizome (score 2); 19%, 24%, and 17% with light discoloration of the rhizome (score 3); and 6%, 0%, and 0% with necrosis of most internal tissues (score 4) (Fig. 4).

All Grand Naine plants used as controls (not submitted to the culture medium containing TDZ and PBZ) presented symptoms of the disease with scores varying from 2 to 5 (Fig. 4A). Therefore, in the first step of the study, eight somaclones from the control treatment, seven from the 0.1 nM and one from the 0.2 mM treatment were selected as resistant, a total of 16 somaclones.

Since plants that were not submitted to the culture medium containing TDZ and PBZ (control) were susceptible to Foc under greenhouse conditions and this did not happen to plants that were submitted to this treatment, it is possible to infer that somaclonal variation was induced in the Grand Naine cultivar. Likewise, Ferreira et al. (2020) obtained banana somaclones of the Prata-Anã (AAB, Pomme subgroup) cultivar resistant to Fusarium wilt by inducing somaclonal variation and testing with the CNPMF 218A isolate, representative of STR4. The plant regulators added to the growth medium at certain concentrations or in combination with other growth regulators, really can affect the rate of somaclonal variation because they increase the process of cell division (Gao et al., 2010).

### 3.3 Confirmation of resistance to Fusarium wilt in selected somaclones

**Fig. 5:**
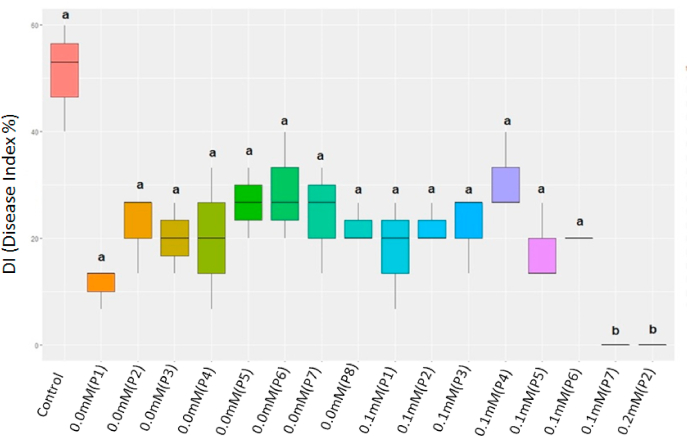
Boxplot of the disease index (DI) of Grand Naine banana plants evaluated in a greenhouse after somaclonal variation induction and selection with fusaric acid for resistance to *Fusarium* wilt. Control: Grand Naine without induction; Control treatment Plant 1 (TCP1) to Plant 8 (TCP8): somaclones without addition of fusaric acid (0.0 mM); Treatment 1: T1P1 to T1P7, somaclones with a fusaric acid dose of 0.1 mM; Treatment 2: T2P1 somaclone with a fusaric acid dose of 0.2 mM. The ID data were transformed into log (x+1). Bars followed by the same letter do not significantly differ by Scott-Knott test (p > 0.05). Embrapa, Brazil, 2021.

**Fig. 6:**
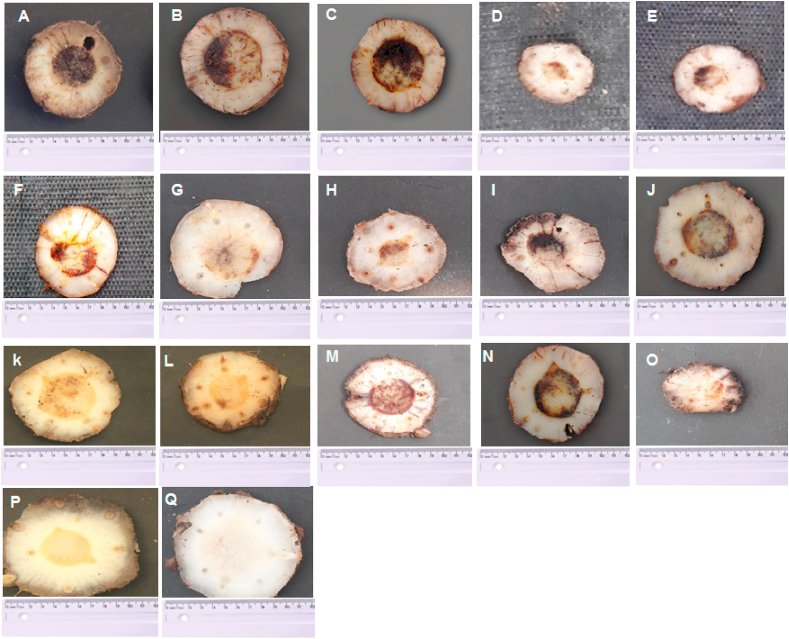
Cross section of a rhizome demonstrating internal symptoms (A to Q). Control (A) Grand Naine without induction, control treatment somaclones (B–I), treatment 1 (J–P), treatment 2 (Q). Evaluation occurred 90 days after inoculation with *Fusarium oxysporum* f. sp. *cubense* in a greenhouse. Embrapa, Brazil, 2021.

Somaclones from the Grand Naine cultivar, which did not present Foc symptoms in the first evaluation, were considered resistant. Hence, were named as follows: eight somaclones from the control treatment (0,0 mM) (T-CP1 to T-CP8), seven somaclones from the FA 0.1 mM treatment (T-1P1 to T-1P7), and one somaclone from the FA 0.2 mM treatment (T-2P1). Somaclones from the control treatment (T-CP1 to T-CP8) and the somaclones from the FA 0.1 mM treatment (T-1P1 to T-1P6, except for T-1P7) were not statistically significant according to the Scott-Knott test for the internal symptom index (ISI) since all of them presented symptoms being susceptible to Fusarium wilt (Fig. 5, Fig. 6A–O). The DI of the somaclones for the control varied from 15% (T-CP1) to 27% (T-CP8); for the 0.1 mM treatment, varied from 0% (T-1P7) and 28% (T-1P4); and the somaclone evaluated in the 0.2 mM treatment had 0% ID assessed in treatment 2 had a DI of 0%. Therefore, the somaclones T-1P7 and T-2P1 were considered resistant to the pathogen with no symptoms for both steps of evaluation and were statistically different from the other somaclones and control (Fig. 5, Fig. 6 P and Q). Hence, both doses, FA 0.1 mM and 0.2 mM, were effective for pre-selection of ‘Grande Naine' banana somaclones resistant to Fusarium wilt.

The probable reason for the plants to resist AF *in vitro* may be associated to the fact that at low concentrations, FA induces plant resistance to *Fusarium* species by promoting plant defense responses (Jiao et al., 2013; Kuanar et al., 2014; Ramírez-Mosqueda et al., 2015, 2019; Samadi and Behboodi, 2006). Some studies suggest that low concentrations of FA trigger various protective responses in plant cells without toxic effects and play a signaling role in pathogen-host interactions (Bouizgarne et al., 2006; Singh et al., 2017). In *Arabidopsis*, the concentrations of FA considered non-toxic were below 10^−6^ M in a cell suspension treatment, which led to the induction of phytoalexin synthesis and rapid responses involved in signal transduction, such as the production of ROS (Bouizgarne et al., 2006).

Treatment based solely on somaclonal variation induction without the addition of FA was not effective for obtaining Grand Naine banana plants resistant to the Foc. This result may be associated with the low number of early multiple shoot clumps used in the *in vitro* multiplication process (35), as most studies use a higher number of plants, considering that the induction of variation occurs at low percentages (Ferreira et al., 2020; Hwang and Ko, 2004).

### 3.4 Staining and clarification of banana roots

**Fig. 7:**
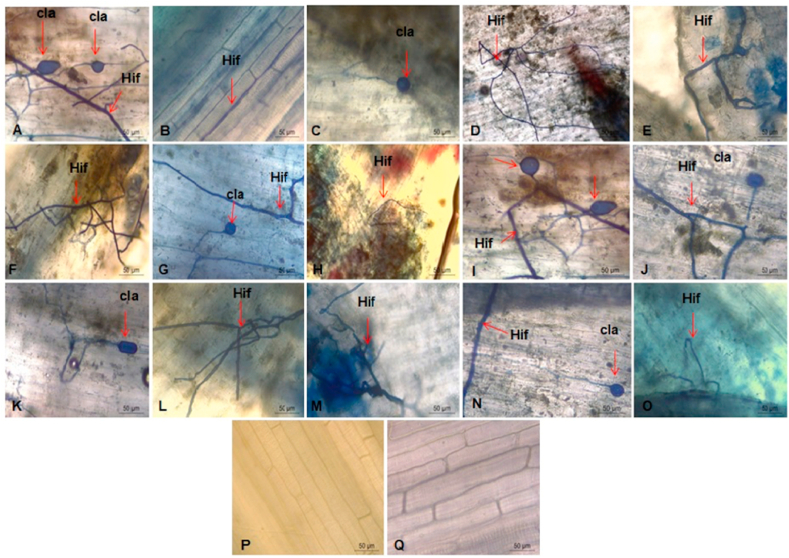
Microscopic image of the root tissue of Grand Naine banana plants after somaclonal variation induction and selection with fusaric acid for resistance to *Fusarium* wilt. Clarification of the roots and vascular structures of the pathogen *Fusarium oxysporum* f. sp. *cubense* with trypan blue. Control (A) Grand Naine without induction, somaclones of the control treatment (B–I), treatment 1 (J–P), treatment 2 (Q). Cla: chlamydospores; Hif: hyphae. Scale: A–Q 50 μm. Embrapa, Brazil, 2021. (For interpretation of the references to color in this figure legend, the reader is referred to the Web version of this article.)

The technique to clarify and stain the roots showed that all the resistant somaclonal variants of the Grande Naine cultivar selected in the first stage of the study presented Foc structures, such as chlamydospores and hyphae, with the exception of the somaclonal variants T-1P7 and T-2P1 (Fig. 7P and Q). The development of hyphae and chlamydospores was seen in the Grande Naine plants used as controls (Fig. 7A).

Therefore, this study shows that genetic variation occurred and that defense responses were activated in the T-1P7 and T-2P1 somaclones, which were based on barriers that are related to pathogen penetration. A similar result was obtained by Ferreira et al. (2020), with the isolate of Foc CNPMF 218A used to select and evaluate resistant Prata-Anã (AAB) banana somaclones; the authors considered the absence of pathogen structures in the root tissues as an indication that the pathogen did not successfully penetrate these tissues, because resistant somaclones probably developed physical and/or chemical barriers to block penetration.

Thus, according to the data obtained in the present study, somaclonal variation induction generates banana somaclones resistant to wilt caused by Fusarium STR4. The somaclones selected as resistant will be tested in the field to validate their agronomic and market potential through a small network of tests conducted under different soil and climate conditions in Brazil. These somaclones are expected to be of potential use for banana producers by providing new crop options available for maintaining the sustainability of banana production. As these resistant somaclones are of the Grand Naine cultivar, if their potential for adoption by banana producing is confirmed, the results can be extrapolated to banana producing countries since this cultivar is the basis of worldwide banana exports market. To our knowledge, this is the first report on somaclones of the Grand Naine cultivar resistant to Foc STR4.

## 4 Conclusions

The development of somaclonal variants of the Grand Naine cultivar is feasible since it was possible to select the T-1P7 and T-2P1 somaclones classified as resistant to Foc STR4 in this study. Two concentrations of fusaric acid are effective for the pre-selection of Grande Naine banana somaclones, namely 0.1 mM and 0.2 mM.

## Declaration of competing interest

The authors declare that they have no known competing financial interests or personal relationships that could have appeared to influence the work reported in this paper.
